# Children’s experiences of meals after obesity treatment: a qualitative follow-up four years after a randomized controlled trial

**DOI:** 10.1186/s12887-022-03387-y

**Published:** 2022-06-14

**Authors:** Nicklas Neuman, Anna Jörnvi, Anna Ek, Karin Nordin, Karin Eli, Paulina Nowicka

**Affiliations:** 1 Department of Food Studies, Nutrition and Dietetics, Uppsala University, Box 560, Husargatan 3, 751 22 Uppsala, Sweden; 2 Department of Clinical Science, Intervention and Technology, Division of Pediatrics, Karolinska Institutet, 141 57 Huddinge, Sweden; 3 School of Anthropology and Museum Ethnography, University of Oxford, Oxford, UK; 4 Division of Health Sciences, Warwick Medical School, University of Warwick, Coventry, UK

**Keywords:** Childhood obesity, Commensality, Family meals, Food environment, School meals

## Abstract

**Background:**

The practice of eating together, *commensality*, is rarely explored in the context of childhood obesity treatment. This is noteworthy given long-standing debates about the physical, psychosocial, and societal benefits of meals, especially family meals. Moreover, as children with obesity experience weight bias and stigma both within and outside the home, it is important to examine meals as a locus of social exchange around food and the body. Our study is based on the premises that eating together (i) matters and (ii) occurs in different environments with diverse social organization, where food-related interactions create varying arrangements of individuals, groups, their statuses, and their actions.

**Method:**

The study explores children’s experiences of meals in different social contexts. Thirty-two children (age 8–10 years) living in Sweden were interviewed, 4 years after they entered an obesity intervention trial. Thematic analysis was applied to the data.

**Results:**

We thematized three meal types, with each meal type having two subthemes: (i) “The family meal”, with “Shared routines, rituals, and rules” and “Individual solutions and choices”; (ii) “The school meal”, with “Rules and norms of the school” and “Strategies of the child”; and (iii) “The friend meal”, with “Handling food that was disliked” and “Enjoyment of food”. These three different meal types carried different experiences of and knowledge about how they were socially organized.

**Conclusions:**

While the children spoke about the family and school meals as meaningful, the friend meal stood out as particularly positive. Contrary to our expectations, the children did not express experiences of weight bias or obesity stigma around meals, nor did they speak negatively about parental control of their food intake. Our findings, especially regarding the friend meal, have implications for further research into commensality and social influences on eating among children with obesity, from early childhood into adolescence.

## Background

The practice of eating together, *commensality* [1–3], is rarely explored in the context of childhood obesity treatment. This is surprising as advice to eat together is part of national dietary guidelines across the world [4–6]. Systematic reviews have demonstrated that family-meal frequency is positively associated with beneficial health outcomes and healthier dietary intakes among children [7–9]. Moreover, a cross-sectional study of children in the United States went beyond frequencies of family meals to look more closely at explanatory mechanisms, identifying an association between positive interpersonal and food-related dynamics (e.g., group enjoyment, relationship quality) during family meals and a lower risk of children having overweight [10].

In addition to supporting healthier eating behaviors, eating together may hold broader psychosocial benefits for children and adults [11–15]. Several qualitative studies further demonstrate that commensality – both within the family and in other social constellations – is often highly valued, desired, and considered worthwhile across cultures [16–22]. For example, Swedish fathers have expressed views of eating and cooking with children, friends, and partners as ways of strengthening social relationships [22], and mothers from the Unites States, of a variety of ethnic backgrounds, have highlighted the family meal as a locus of family togetherness, communication, and joyful interaction [16, 17, 19]. Shared (family) meals are thus considered to form cohesive norms and healthy routines that regulate eating behavior in beneficial ways [2].

One drawback of the literature on children’s commensality and health is that it is dominated by observational, primarily cross-sectional, studies. This methodological problem is aggravated by a lack of general agreement on how to assess the social functions of eating together [23]. As a result, reports of various effects of eating together on, for example, different populations’ body weight, food habits, or psychosocial well-being have been criticized for not being able to determine causality [24–26].

The qualitative literature further reveals that food and eating within families’ everyday lives are not always positive [27]. Commensality at home may be associated with conflict, guilt, and shame [28], as well as memories of economic hardship and abusive behavior, such as hurtful comments about children’s bodies [21]. Berge et al. also reported that negative family dynamics such as hostility, parental permissiveness, and inconsistent discipline strategies regarding food were associated with a higher risk of overweight [10]. Still, the potential of commensality to cause emotional harm is not addressed adequately in the childhood obesity literature. This persists despite the stigmatization of obesity across many cultures [29–31] – a stigmatization that often infiltrates the home [32, 33], with family meals being a key occasion where obesity stigma may be expressed [34]. Thus, it is relevant to understand how children who have participated in obesity treatment from a young age perceive meals in different food environments. In treatment, a goal is to avoid food and meal situations becoming negatively charged, but there is scarce knowledge about this.

An additional gap in the literature is that it largely overlooks heredity and the multiplicity of environments in which children eat. Adopted children’s body mass index (BMI) is correlated more with the BMI of their biological families and less with the BMI of their adoptive families, while twin studies suggest that the effect of shared environments (e.g., the family and shared social circles) may peak in early childhood [35]. Thereafter, genetic heritability of both eating behavior and BMI become increasingly dominant, and from the early teen years they are explained primarily by genes and unshared environmental factors [36]. Children are thus exposed to a variety of environments in which food is involved, many of them beyond parental control.

Based on the premises that eating together (i) matters and (ii) occurs in different environments with diverse social organization, we explore children’s experiences of meals in different social contexts. By social organization, we refer to a product of social interaction, which leads to varying arrangements of individuals, groups, their status, and actions within different settings. For example, any given child has a certain status as son/daughter within a family or as a pupil within a school, which results in social expectations of proper behavior, limits to what one is allowed to do, division of responsibilities, and so forth. In this study, we analyze interviews with children (age 8–10 years) living in Sweden, 4 years after they entered an obesity intervention trial that involved preschool-age children and their families. In this qualitative study we focus on the children’s experiences and perceptions of meals in a variety of social contexts. In an earlier qualitative investigation conducted soon after treatment, we found that the children’s parents worried about the social stigma attached to obesity, citing social eating situations as particularly fraught [37]. Four years after the treatment, participating parents expressed struggles with maintaining children’s healthy routines, especially outside the home, for example with friends, grandparents, or in school [38]. They also spoke of social comparisons due to the children’s body shapes, for example, comparisons arising from different abilities when engaging in sport or having a sibling with normal weight, but also weight-based teasing from other children in school. In a second publication from the 4-year follow-up interviews, the parents expressed additional concerns about changing the child’s food environment at home, including worries about denying a hungry child food, fears of future eating disorders, or worries about eating habits in other food environments [39]. In this study, we turn to the children for their perspective, in an attempt to understand whether and how issues related to obesity entered into their social eating interactions.

### Theorizing meals and eating together

When analyzing the interviews with the children, we define a meal as a regularly occurring and socially organized eating event, distinguishable in time and space, in which the eating activity is at the center (rather than secondary to drinking or to another activity) [40–42]. Eating together is not confined to sharing a meal, and may involve splitting a bowl of snacks while watching a movie, grabbing an ice cream while out on a walk, or taking a snack break together during sports practice. However, in this article, although between-meal snacking is discussed, the focus is on shared meals.

In some social scientific accounts, the shared meal is emphasized as a means of social order and communion [2, 43, 44], something that bonds us together through collective rules, rituals, norms, and values. In others, the meal’s role in social exclusion and hierarchy is highlighted, demonstrating how domestic commensality often depends on unequal (primarily gendered) divisions of work and responsibility [27, 45–47]. From an evolutionary point of view, food sharing is a central aspect of human cooperation and reciprocity [48, 49]. This makes the meal a venue for social exchange, not only between adults and their children but also between adults and non-kin children, among adults, and among children. Non-human animals share food and eat in groups too, but the deeper social norms around eating and the cultural symbolism attached to food seem to be distinctly human [50]. Viewed from this perspective, eating together is likely a fundamental part of our social nature [51, 52].

We thus acknowledge the meal as an influential part of how human life is organized through social interaction, beginning in childhood. Qualitative studies have portrayed family meals as both socially and nutritionally positive [53, 54], and school meals as opportunities for children to socialize with friends [55, 56]. While these studies contribute to the literature in meaningful ways, especially by illuminating the children’s own perspectives, they do not include non-family environments beyond schools and do not highlight the experiences of children with overweight or obesity. In this study, we examine mealtime sociality in several environments that are part of children’s lives and focus on children who have undergone treatment for obesity. As mentioned above, their parents expressed a great deal of concern [37–39]. In part, parental concerns focused on children’s eating habits outside of the home. However, parents were also concerned about their children’s bodies and appetites being different from those of other children, and worried their children might suffer due to these differences. Were these concerns present in the children’s own stories – and if so, did they influence how the children experienced mealtimes?

### Aim

This study explores children’s experiences of meals in different social contexts, 4 years after the start of obesity treatment.

## Method

### The study setting

In 2012—2016, the More and Less Study (ML), a randomized controlled trial, was conducted to examine the effectiveness of a parent support program as treatment for preschool-age children with obesity compared to standard treatment as offered in an outpatient pediatric clinic. The children lived in Region Stockholm, Sweden, and were recruited continuously during the study’s 4 years, mainly from primary healthcare [57, 58]. The following inclusion criteria were applied: children aged 4–6 years, with obesity according to age- and sex-specific international cut-offs [59], lack of other conditions or diseases that could affect the child’s weight and height, and parents’ ability to understand and communicate in Swedish [57]. The results showed that after 12 months, children whose parents participated in the parent support program delivered in groups had significantly improved their weight status compared with children who received standard treatment (i.e., individual visits to primary health care, attended by the child and one of his or her parents) [58].

Four years after the initiation of treatment, children and parents were invited to an interview study. We aimed to conduct 30 interviews which, based on our previous studies [37, 60] and established criteria for thematic analysis [61], was judged as a reasonable sample size for reaching saturation. To account for drop out, all families who were recruited during the first 2 years of the study (*n* = 67), were invited. We were unable to get in contact with 14 families, 19 declined, and two had moved abroad.

Thirty-two children (17 girls and 15 boys), age 8–10 years old, were interviewed. Twenty of the interviewed children were from the intervention group (their parents participated in the parent program) and 12 were from the control group (that received standard treatment). Compared to the children who were not interviewed, those who were had a significantly lower weight status at baseline and at the 12- and 48-month follow-ups. They also had a larger reduction in BMI z-score at the 12-month follow-up, but not after 48 months. At both follow-ups the majority were still classified as having overweight or obesity. However, compared to children who were not interviewed, more of the interviewed children had overweight; this difference was not statistically significant at the 48-month follow-up. Among the parents of the interviewed children, about half had foreign background and about half had a university degree. In the families who were not interviewed, more fathers were classified as having overweight or obesity. We provide a descriptive summary of the children’s and parents’ data in the 32 families who were interviewed (Table 1).

**Table 1:** Characteristics of children interviewed 48 months after obesity treatment was initiated and their parents' characteristics at baseline

***Child***	***(n = 32)***
	*n (%)*
Treatment group	
Parent group	20 (63)
Standard treatment	12 (37)
Gender	
Girl	17 (53.1)
Boy	15 (46.9)
Weight status *(baseline)*	
Overweight	8 (25.0)
Obesity	24 (75.0)
Weight status *(12 months)*	
Normal weight	1 (3.1)
Overweight	15 (46.9)
Obesity	16 (50.0)
Weight status *(48 months)*	
Normal weight	1 (3.1)
Overweight	13 (40.6)
Obesity	18 (56.3)
	*Mean (SD)*
Age (years) *(48 months)*	9.4 (0.8)
BMI z-score *(baseline)*	2.8 (0.6)
BMI z-score *(12 months)*	2.4 (0.8)
BMI z-score *(48 months)*	2.4 (0.6)
Mean change in BMI z-score between baseline and 12 months	−0.41 (0.5)
Mean change in BMI z-score between baseline and 48 months	−0.38 (0.5)
***Mother***	***(n = 30)***
	*Mean (SD)*
Age (years)	36.6 (4.7)
	*n (%)*
Foreign background	
Yes	18 (60.0)
No	12 (40.0)
Weight status	
Normal weight	12 (37.5)
Overweight	8 (26.7)
Obesity	10 (33.3)
University degree	
Yes	15 (50.0)
No	15 (50.0)
Employment	
Full-time	9 (30.0)
Part-time	8 (26.7)
Student	2 (6.7)
Parental leave	7 (23.3)
Unemployed	4 (13.3)
Income (SEK/month)	
< 10,000	5 (17.2)
10,000 < 20,000	18 (62.1)
20,000 < 30,000	3 (10.3)
30,000 < 40,000	3 (10.3)
***Father***	***(n = 29)***
	*Mean (SD)*
Age (years)	39.6 (7.7)
	*n (%)*
Foreign background	
Yes	15 (51.7)
No	14 (48.3)
Weight status	
Normal weight	7 (24.1)
Overweight	11 (37.9)
Obesity	11 (37.9)
University degree	
Yes	12 (41.4)
No	17 (58.6)
Employment	
Full-time	25 (86.2)
Part-time	1 (3.4)
Student	1 (3.4)
Unemployed/sick leave	2 (6.9)
Income (SEK/month)	
< 10,000	5 (17.2)
10,000 < 20,000	8 (27.6)
20,000 < 30,000	12 (41.4)
30,000 < 40,000	3 (10.3)
40,000 < 50,000	1 (3.4)

Abbreviations: *SD* Standard deviation, *BMI* Body mass index

Children’s weight status was classified as normal weight, overweight, or obesity according to age- and sex-specific international cut-offs for children. Foreign background: parent and both grandparents born abroad, or parent born in Sweden and both grandparents born abroad. Among the parents with foreign background, the average time spent in Sweden were 23 and 21.6 years for mothers (*n* = 12) and fathers (*n* = 11), respectively. The parents’ weight status was classified as normal weight, overweight, or obesity according to the World Health Organization’s reference values for BMI. Parental characteristics are baseline values. Income data is missing for one mother

### Development of the interview guide

An interview guide was developed by a multidisciplinary group of six researchers with extensive experience in research and clinical work with children. To ensure the validity of the interview guide, the researchers evaluated each question in the interview guide individually by using Content Validity Index (CVI). To assess the CVI for the interview guide, the group filled in a form judging the relevance of a particular body of items. This process allowed to assess the relevance of questions in relation to the concepts that the interview guide intended to capture [62].

The preliminary interview guide was pilot tested with nine children (eight girls and one boy), six to 13 years of age. The pilot resulted in some revisions, such as reordering and reformulation of questions. The final interview guide included four subject areas that the multidisciplinary team of experts deemed relevant when talking to children with obesity, based on their research and professional experience: 1) food preferences (including selective eating); 2) experience of hunger, satiety, and cravings; 3) experience of meal situations; and 4) leisure activities. The order of questions was based on the sensitivity of the subject. For example, questions about comparisons with other children’s eating appeared in the middle of the interview and questions about leisure activities appeared last [63]. This allowed the interviewer to build trust with the child before asking potentially sensitive questions [64]. The interview ended on a topic that from clinical experience was perceived as less sensitive to talk about.

### Conducting the interviews

The interviews were conducted approximately 4 years after the children entered the obesity treatment trial. Interviews took place from February 2017 to December 2018 and were conducted by KN, a licensed pediatric nurse with 30 years of experience, MSc in Nursing, and additional training in qualitative methods. The interviews were conducted in the same setting the children had previously visited (e.g., the research center, a primary health care center, or an outpatient pediatric clinic) with at least one parent present (although not partaking in the conversation). Interviews lasted between 11 and 51 minutes (25 minutes on average). They were audio-recorded and transcribed verbatim by the pediatric nurse (KN) and a registered dietitian with previous training in transcribing as a journalist.

### Data analysis

The interviews culminated in 119 A4 pages of transcription, treated as one unit of material, meaning that the experiences of the children in the intervention group were not separated from those of the children in the control group. The main reason for not separating them is that the interview guide had very broad questions about the children’s everyday lives, including topics that were not directly connected to the outcomes of the trial as such. The data were initially analyzed thematically [65, 66] for AJ’s master thesis. NN, who was not involved in the thesis supervision, reanalyzed the data, still in accordance with the principles of thematic analysis, to ensure rigor, validity, and consistency. Both NN and AJ were blinded to which groups the children had belonged to in the trial, so they were not biased by knowledge of a given child’s treatment when analyzing his or her responses in the interview. They found no indication that responses differed systematically in a way that can be attributed to the different treatment approaches in the RCT.

An initial open coding process resulted in a preliminary scheme and preliminary themes which were checked and modified by AJ and then discussed between NN, AJ, and PN. Following this discussion, NN, AJ, and PN decided to focus the analysis on the context of meals described by the children, thus connecting eating events to the children’s talk about social interactions. Following the decision to focus on meal types, the coding had to be revised to some extent. NN recoded the data with a focus on meal types, in close collaboration with AJ and in regular discussions with PN. AJ also recoded three interviews to calibrate the analysis. Inter-coder reliability was not calculated, but the coding was discussed for consensus and modifications. This analysis, too, was subject to collective deliberation. Following the data recoding, NN and AJ discussed the abstraction of relevant codes to categories and themes. They created a document for the larger author group (including KN, the interviewer) to discuss, in which the suggested themes were presented together with several quotes representing the analysis. The author group discussed the analysis until a consensus was reached. The outcome of this structured and collective process of analysis was the identification of three meal types, in which the children expressed awareness of the distinct organization involved and their own perceptions of the food and the meal situation.

## Results

**Fig. 1:**
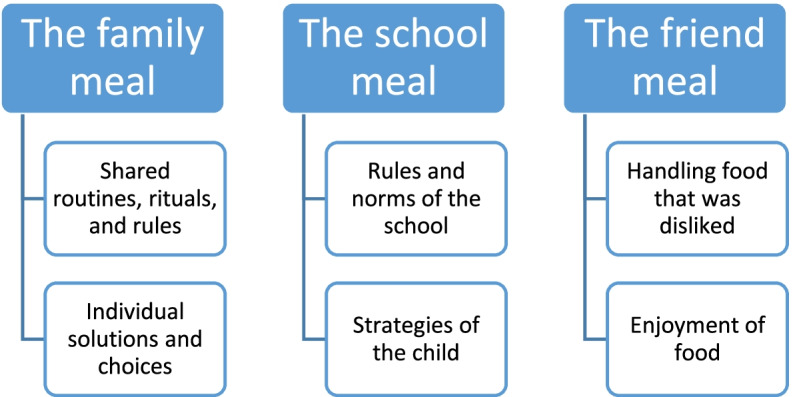
Themes (meal types) and subthemes (social organization of the meal types)

Children spoke about many forms of meals, such as everyday dinners, special dinners, and picnics. We chose to divide our results into three main meal types, defined by who took part in them: the family meal, the school meal, and the friend meal (which we define as a meal with a friend at the friend’s home, or a meal with a friend invited to the interviewed child’s home) (Fig. 1). All names are pseudonyms. In the quotes, we use bold text to indicate the researcher’s questions and non-bold text to indicate the child’s responses.

### The family meal

The family meal featured centrally in the interviews, as did food within the family more generally, including children’s domestic food chores and other eating events (e.g., between-meal snacking). In general, the children expressed ideas about shared routines, rituals, and rules of their families, from the activities and norms of everyday life to special occasions. But they also spoke about flexibility, where rules could be stretched, particular choices made, and issues solved by the individual child.

### Shared routines, rituals, and rules

By *routines* we refer to the regularly occurring patterns of ordinary activities, and by *rituals* we refer to special activities associated with certain symbolic meanings (e.g., birthdays or weekends). Some routines implicated the social composition around shared meals. For example, there could be differences depending on which parent a child lived with on a given week (when parents’ custody was shared) while some children spoke of meals including all parents and siblings. Emma said her family mostly shared meals with their grandmother:

**Who usually joins then? Is everyone sitting around the table like this? Mom and dad and** ... Then almost everyone is sitting around the table and grandma, she … lives on the fifth [floor], so she usually comes down to us and eats. But dad usually doesn’t, he usually eats after us. Sometimes mom does, otherwise the kids usually eat first and then mom and dad. (Emma)

Connected to the social composition around the meal were the routines of places where the meals were eaten. Children described household rules about where eating was or was not allowed, with the kitchen table being the usual place where children had their main meals. Emma and Ebba said they were not allowed to eat on the sofa, exemplifying awareness of explicit rules set by parents. More commonly, however, children described flexibility in eating spaces, for the family as a collective, for a certain family member, or for the interviewed child. Like Josef:

In the living room and in our room we usually [sit and eat]. **In your room?** Yeah. **Where do you prefer to sit? Do you think that** … In my room. **In your room, what do you do then? Do you do anything [else] while eating?** I watch and put on a movie. (Josef)

David expressed the opposite preference, saying that his family’s sporadic living room meals were cumbersome due to the extra work involved in moving things to and from the kitchen. Gabriel’s whole family had their Friday dinner in front of the TV, while Edith’s family did the same thing when watching movies, although she said that the best place to have dinner was her room. Thus, certain places to eat also implied solitary meals for the children. In addition, families’ eating spaces could include venues outside the home. August’s, Saga’s, and Edith’s families went to restaurants on weekends, and Märta said she enjoyed outdoor summer picnics with her family.

Children demonstrated an awareness of how places were connected to everyday routines and special occasions. When special occasions were described, children also spoke about the foods eaten. Maja was allowed sweets and snacks on Saturdays, and Saga’s restaurant visits included “hamburgers and stuff” as well as “rice and meat and stuff.” Märta said family picnics included barbecues, as well as “salad and donuts and stuff.” Alma’s parents normally decided what to eat, but she was allowed to decide with her sister “on Fridays for example.” This was the case on the day of the interview:

**Who decides what you should eat?** Mom and dad. Sometimes my sister and I get to choose what we want a bit. **When do you usually get to do that?** Don’t really know, on Fridays for example. **Are you going to decide today then or?** Today we have already decided … **What will it be then?** It’s going to be burgers. … **But otherwise on Wednesdays and so on, mom and dad decide?** Yes. **Do you think that’s good?** Yes. **That mom and dad decide or did you want to decide?** Well then I don’t know what I would have chosen. (Alma)

The children described how their eating (e.g., what to eat or portion sizes) could be restricted by their parents. Sometimes children also mentioned following food rules for reasons other than their own way of eating, like Gabriel who said he could take seconds as many times as he liked, “but one has to save for mummy’s can [lunch box] for work.” Taken together, the social compositions of meals and places to eat could vary, but were described as highly regularized, while the children also expressed awareness of several limits set by their parents on how and what to eat, depending on context.

### Individual solutions and choices

When faced with food situations that did not follow the norms and routines of the family, children made a variety of individual solutions and choices. This included situations where they were served food they were unsure of or disliked, as well as situations where they were hungry and wanted extra food, either second servings of a meal or snacks between meals.

In addressing such situations, the children were aware of the need for negotiations or other solutions. When served a new food, Alma said she first tasted it to see if it was acceptable, and if not, she chose *filmjölk* (a fermented dairy product similar to yoghurt). Several children described similar practices, trying out new foods first, and then choosing a snack if they did not enjoy them. Oliver described another solution: “I’m giving it to dad, he eats everything.” When further asked about how he handled his own hunger after giving his food to his father, he said:

I’ll take something from the fridge. **Do you have to ask then before you get to take from the fridge, or?** Yes. **Do you ask mom or dad?** Both. **What do they say then?** Eh, they usually say yes. **Do they decide what you’re allowed to take then?** Ehum, I usually take whatever, some things have to be left for a party or something. (Oliver)

As the quote shows, Oliver expressed a certain degree of freedom to make his own choices, but not without his parents’ say. Some children recalled other strategies to deal with unwanted food such as going out to buy something else to eat, just tasting the food, or eating less. However, even though the children usually expressed some level of flexibility in the home, exceptions existed, such as Albin who claimed to eat whatever his mother served even if he disliked it.

When it came to having seconds, most children said they had to ask for more, sometimes successfully and sometimes not. Ebba, quoted below, expressed this clearly, and also pointed to her parents limiting her portions:

**What do you do if you are hungrier then, if you have received a portion from mom or dad?** Then I ask if I can take some more and then they can say no or yes. **What’s the reason, when do they say yes and when do they say no?** It depends on whether I have taken a small or a large portion or if I’m very hungry. (Ebba)

### The school meal

As in stories about the family meal, when children spoke about the school meal they also focused on rules and social norms, as well as on strategies to handle food they did not enjoy. In Sweden, the school lunch is free of charge. It is also legally required to be nutritious [67] and recommended to involve a variety of different foods [68, 69]. This is relevant contextual information for two reasons. First, school lunches are not restricted by the financial resources of children’s families, and the same food is accessible to all children in any given school. Second, we can assume that the participating children were exposed to a variety of foods provided based on nutritional considerations.

### Rules and norms of the school

When speaking about school meals, the children focused on the formal and social rules that organized them. Gabriel described restrictions during the school meal, such as the number of pancakes each child was allowed. Likewise, Saga said a child could have seconds, unless “one is a little too late.” Eating for too long, until other classes came to the canteen for their lunch, also meant insufficient time to finish, thus “we usually scrape it off” (i.e., scrape off food into the waste bin). This exemplifies a difference between the family meal and the school meal, in that the time devoted to the meal was decided in relation to formal schedules. The children also expressed obeying stricter norms in school compared to the family meal. Edith stated explicitly that throwing away food makes “the ones cooking the food really angry” and Ali stated that “you’re not allowed to throw away sometimes … you have to eat up.” Some children also mentioned being assigned chores around the meal such as cleaning the table. Saga said that the teachers demanded that the children eat vegetables, and that the children were assigned chores (e.g., cleaning the tables) as “class hosts.” Thus, the children were clearly aware of several aspects of social expectations around their meal behavior, including explicit demands and strict norms. When behavior was less regulated, however, the children had a lot to say about their own strategies.

### Strategies of the child

The children spoke of specific strategies they used when the school meal included foods they disliked. Elin said she would “usually just eat vegetables, I usually try a bit … [and] if I don’t like it, I usually don’t take that much, just a tiny bit that I throw away.” Saga described the same strategy – eating vegetables and then throwing the food away – while Märta had a sandwich instead. Alicia, furthermore, said that she usually tried a bit; then if the food was good she would take seconds and if not she would throw it away. Hailey’s description further exemplifies the connection to the previous subtheme. She could not remember any time she disliked the food at home, “but it’s more like in school then it’s like ‘Ooh, I won’t like this’,” further describing that:

… I take a little and taste, because then I take like a little bit and lots of vegetables and a sandwich instead … Then I usually, like, take a really small bite and then I usually taste but then I usually don’t like it, but you always have to take something. (Hailey)

Thus, Hailey experienced rules (or at least expectations) for the children to have to try something, echoing the previous subtheme, as well as her ways of handling this. Two other children, Ebba and Gabriel, said that they would eat even if they disliked the food. But not much. “**[A]t someone’s home then?**”, the interviewer asked Ebba: “Then I can eat a little.” “**[A]t school then?**”, she continued: “Then I also eat a little if I don’t like it.” Lastly, when Gabriel was asked about what he does when the food is not very good, the following conversation emerged:

I eat anyway because I have nothing else to eat. **At school then?** Then I have to eat that or something else, like yesterday I think it was … I took, so it was food that I don’t like so I just took some chicken with some sauce. **Is that what you usually do, there’s always at least something that you like, that you can eat?** This is the first time I’ve done that, so if the food isn’t good I will eat it anyway. ... **If you’re invited over then, to some friend’s home or?** Then I eat the food I don’t like. (Gabriel)

### The friend meal

Unlike discussions of the family and school meals, which revolved around established rules and routines, discussions of the friend meal carried different associations, foregrounding the thoughtful handling of food and enjoyment of eating.

### Handling food that was disliked

The children were sometimes asked what they did if they did not like the food at a friend’s house. To this, Saga replied that she “would dare to say that I don’t like it and that I can do something else while they eat.” However, she is an exception to what was a clear pattern. More commonly, like Gabriel in the last quote of the previous theme, children said that even if they did not like the food, they ate it, or they lied about being full. Emma said that she did not want to eat if she disliked the food at home, but that if the same situation occurred at a friend’s home she would eat, “even if I don’t like it that much ... Like, I don’t know, I usually don’t say that I don’t like it because I eat anyway.”

Ebba, Anton, Albin, and Märta described acting similarly. Moreover, Märta exemplified how some children explicitly mentioned that refusing food may be socially awkward or make the host feel bad. When asked what she does if the food does not taste good at a friend’s home, she replied: “I don’t say anything because that would be embarrassing if [my friend’s] parents were there, so I don’t say anything.” Hailey also said that she tried foods for politeness, though she tried to avoid eating large portions: “not [to say], like, ‘Eew, I don’t like the food’, but, and then be impolite but, like, yeah, and then maybe I haven’t eaten everything.” Likewise, Ibrahim pretended to eat if he did not like the food, and would “try to swallow immediately … without chewing so much.” He continued to talk about this, and was then asked:

**Why do you do that then?** Because, if you like, if you don’t like how something tastes, or how it feels, and then you just want to remove it ... **Yes, I was thinking, like, do you think that the one who cooked can get sad, or why do you eat anyway?** You can get a little sad, why did you make the food if the person you made it for doesn’t want it? (Ibrahim)

### Enjoyment of food

In general, the children enjoyed the food at their friends’ homes. There were two categories of expressions about the food in friends’ homes. The first comprised simple statements about not having had experiences of the food ever being bad. The second included more explicit expressions of liking the food, emphasizing the joys of eating things they were not used to, or saying these foods were as good as the ones they usually ate.

Examples of the first were Alma’s and Alva’s replies to the same type of question discussed in the previous subtheme, about what they do if they are served something they dislike at friends’ homes. “I don’t know, it has never happened,” said Alma. When further probed if Alma’s friends’ parents were good cooks she said “Naw, they probably cook just as good as my mom.” Alva also said that such a thing had never occurred, and was also probed about the reverse situation, if a friend disliked the food in Alva’s home: “I don’t know, that’s never happened. **How lucky**. Yeah, mom is a good cook.” As the quotes show, neither child could even think of an example since their friends’ parents were seen as equally good cooks as their mothers.

The second category was exemplified by Elin who said that meals at friends’ homes were “usually good, because it’s usually not what we have at home” and Bella who said that “it’s always good, at friends’ [homes], it’s always good food … especially at [friend’s name].” August’s “second best friend” was half Swedish, and the national cuisine cooked by his friend’s non-Swedish parent was something August loved, “almost more than my favorite dishes.” Alicia was vegetarian, but that did not seem to be a problem when eating at her friend’s home:

**Do you ever eat at a friend’s house?** Yes, but they know I’m a vegetarian so they … my best friend, when I come home to her, her mother made salmon once and small pancakes and then when I came home to her dad then he made, what’s it called, vegetarian burger**. Is it always food you like when you’re away?** Mmm. **And when she comes to your house then?** But, then like, she can eat anything so she can eat the food we eat. **Does she like it then?** Yeah. **Mom is good at cooking?** Yeah like, some, I eat meat at home, it’s vegetarian meat … they have come to my house and eaten meat at my house, but then we say vegetarian meat, not like real meat. (Alicia)

While there are no explicit references to the meal’s function in social relationships, all quotes here, and more that were similar, at least make it clear that the friend meal was enjoyable, whether the children ate at friends' homes or invited friends home.

## Discussion

This study has explored experiences of meals among preadolescent children, 4 years after they started obesity treatment. We found that children spoke of mealtime experiences related to three distinct meal types: the family meal, the school meal, and the friend meal. A striking and key finding was that obesity or weight did not enter into the children’s discussions of any meal. But clear differences emerged between children’s experiences, the type of meal, and its social context. All meal types and contexts involved a mixture of perceived or explicit rules from the adult world and individual flexibility among the children themselves, with distinctive awareness of when and why the children thought they could make individual adjustments. Also, the friend meal was described in almost unanimously positive words, unless the child was explicitly asked about disliked foods.

The interviews provide a glimpse into how children with overweight and obesity handle meals and different food environments. Arguments in favor of family meals’ nutritional health benefits build on assumptions about healthy socialization and exposure to healthier foods within the family. Maybe this is true, but the family meal is complex; according to the children, it is subject to different routines, rituals, and rules depending on context (e.g., everyday regularities or special occasions). Second, the meaningfulness of different meal contexts points to the importance of broadly mapping out children’s food environments beyond the family, especially when considering the behavioral-genetic evidence suggesting that other environmental factors dominate from around 12–13 years of age [35, 36]. This point is also relevant regarding between-meal snacking. Even though this was not analyzed in-depth here, it was indeed talked about among the children. Unhealthy snacking beyond parental control was also a concern raised in the parent interviews [39]. As such, it must be considered an important contribution to children’s nutritional intake and their social eating, and something for future studies to explore more carefully.

Compared to the family meal and the school meal, which have already been the focus of research, prevention, and treatment, we find children’s descriptions of the friend meal particularly thought-provoking. While this meal type has hitherto been largely overlooked, it stood out in the children’s interviews. They reported enjoying food at their friends’ homes, but also being attentive to eating what was served. This is of relevance, first, for the understanding of dietary habits and weight management from early childhood to adolescence, when a diversity of social contexts provides opportunities to eat without parental involvement. Second, it matters for the continued understanding of the social functions of eating together among children and adolescents, beyond the contexts of the family and school, that are otherwise commonly studied. In fact, adolescents’ eating with friends outside the family environment has been associated with consumption of ultra-processed foods [70], while their friendship ties are associated with both unhealthy food habits [71] and body weight [72]. In sum, as children grow and mature their parents have less and less influence over their eating habits. Moreover, comparing how the children described family meals and friend meals, it is interesting that friend meals were described so positively. In the cross-sectional study by Berge et al. [10], a positive climate around the home meal was associated with a lower prevalence of overweight in the children. Our findings suggest that children’s positive experiences of the friend meal should be researched further, and may usefully inform future interventions into family meals.

Returning to the worries expressed by the parents in previous publications, about the children feeling different, being stigmatized, or being bullied, [37–39], these did not emerge in the children’s interviews. No child expressed feelings of being different or ill-treated. On the contrary, the children responded in ways that seem typical of children in general. This is interesting in and of itself, since experiences of feeling different or being ill-treated were a plausible expectation. This lack of expressions connected to weight bias or stigma may seem surprising, but could be explained by several factors. One possibility is that the children were indeed free from weight bias and stigma in the meal context, and that the meal was an element of the children’s life that they generally enjoyed. It could also be explained by the children’s young age (prepubertal) combined with how the questions were asked. The children may not have made the connection between body size and meals because the interview guide was not developed to specifically ask about stigma. A direct question about whether they were being teased or bullied because of their appearance may have provided us with different information. However, since we judge such an approach to be unjustified from an ethical point of view, we suggest that future studies be designed to capture such phenomena indirectly, for example through ethnographic or experimental studies of children’s meal situations, preferably in samples that allow for comparisons of children with a diversity of body sizes and shapes. Another explanation could be that because all children and their families took part in obesity treatment, the parents were well-equipped to prevent bullying in their children’s peer group, as well as boost their children’s self-esteem and help them handle obesity stigma.

In childhood obesity treatment parents are often supported in how to set up rules and routines for appropriate portion sizes, healthy food choices, and food situations. Thus, we assume parents in both the intervention group and the control group of the ML trial received this support. We found that children were aware of parents’ (and school staff’s) attempts to limit their portion sizes and decide what food they ate. They did not talk about their parents’ control of their eating in negative terms [21], but rather as a matter of fact, suggesting that this was not a sensitive topic to discuss. Since we only interviewed a small group of children with overweight or obesity, we do not know if most children of this age, regardless of weight, would describe meal situations and foods in a similar way.

It seems likely that many of the children’s solutions and strategies – such as eating fruit, vegetables, or yogurt when hungry between meals – are indirect evidence of how adults around the child managed to communicate healthy eating habits without focusing on the child’s weight. This association did not clearly come out of the interviews. However, this could be a methodological artefact since the interviewer did not ask the children if their parents tried to restrict their food intake due to weight concerns. Additionally, due to ethical considerations, a parent was always present during the interviews, and it is possible that children avoided discussing their parents’ practices negatively, such as describing overly restrictive food control. On the other hand, the presence of a parent could also be a strength in helping the child feel more relaxed around the interviewer.

The main strength of this paper is the sample – children who have had obesity at a very young age – a vulnerable population about whose experiences of meal situations we have very little knowledge. As already mentioned, the sensitivity of the issues covered required methodological choices that may have limited our data, yet we believe that the advantages of giving voice to the children directly outweigh the methodological problems, and we have no indications that the interviews harmed them. Other strengths are the systematic and in-depth analysis involving several authors at different stages, the careful and systematic development of the interview guide, and the professional competence of the research group in qualitative methods, pediatrics, and the sociology of food.

There are several implications for further research based on our findings. There is a gap in the literature on commensality and social influences on eating that focuses on children with overweight and obesity, despite its relevance for understanding the social and nutritional significance of different food environments. This includes both early childhood and adolescence, since the relationship to food and the body changes as the child grows. As mentioned above, weight-related bias and stigma should be considered in those studies, but approached in ways that can capture such phenomena indirectly, as to reduce the risk that the research itself harms the child.

## Conclusions

In exploring experiences of meals in different social contexts among preadolescent children, who had started obesity treatment 4 years earlier, we identified how three different meal types – the family meal, the school meal, and the friend meal – carried different experiences of and knowledge about social organization. While the family and school meals were indeed meaningful to the children, the friend meal stood out as particularly positive. Contrary to our expectations, the children did not express experiences of weight bias or obesity stigma around meals or parental control of their food intake. Our findings, especially regarding the friend meal, have implications for further research into commensality and social influences on eating among children with obesity, from early childhood into adolescence.

## Data Availability

The data used and analyzed for this article are available from Paulina Nowicka, the principal investigator, on reasonable request.

## Abbreviations

BMI: Body mass index
CVI: Content Validity Index
ML: The More and Less Study
MSc: Master of science
